# The role of machine learning in advancing diabetic foot: a review

**DOI:** 10.3389/fendo.2024.1325434

**Published:** 2024-04-29

**Authors:** Huifang Guan, Ying Wang, Ping Niu, Yuxin Zhang, Yanjiao Zhang, Runyu Miao, Xinyi Fang, Ruiyang Yin, Shuang Zhao, Jun Liu, Jiaxing Tian

**Affiliations:** ^1^ College of Traditional Chinese Medicine, Changchun University of Chinese Medicine, Changchun, China; ^2^ Department of Encephalopathy, The Affiliated Hospital of Changchun University of Chinese Medicine, Changchun, Jilin, China; ^3^ Institute of Metabolic Diseases, Guang’anmen Hospital, China Academy of Chinese Medical Sciences, Beijing, China; ^4^ Department of Hand Surgery, Second Hospital of Jilin University, Changchun, China

**Keywords:** diabetic foot, machine learning, diabetes foot ulcers, precision medicine, computational pathology

## Abstract

**Background:**

Diabetic foot complications impose a significant strain on healthcare systems worldwide, acting as a principal cause of morbidity and mortality in individuals with diabetes mellitus. While traditional methods in diagnosing and treating these conditions have faced limitations, the emergence of Machine Learning (ML) technologies heralds a new era, offering the promise of revolutionizing diabetic foot care through enhanced precision and tailored treatment strategies.

**Objective:**

This review aims to explore the transformative impact of ML on managing diabetic foot complications, highlighting its potential to advance diagnostic accuracy and therapeutic approaches by leveraging developments in medical imaging, biomarker detection, and clinical biomechanics.

**Methods:**

A meticulous literature search was executed across PubMed, Scopus, and Google Scholar databases to identify pertinent articles published up to March 2024. The search strategy was carefully crafted, employing a combination of keywords such as “Machine Learning,” “Diabetic Foot,” “Diabetic Foot Ulcers,” “Diabetic Foot Care,” “Artificial Intelligence,” and “Predictive Modeling.” This review offers an in-depth analysis of the foundational principles and algorithms that constitute ML, placing a special emphasis on their relevance to the medical sciences, particularly within the specialized domain of diabetic foot pathology. Through the incorporation of illustrative case studies and schematic diagrams, the review endeavors to elucidate the intricate computational methodologies involved.

**Results:**

ML has proven to be invaluable in deriving critical insights from complex datasets, enhancing both the diagnostic precision and therapeutic planning for diabetic foot management. This review highlights the efficacy of ML in clinical decision-making, underscored by comparative analyses of ML algorithms in prognostic assessments and diagnostic applications within diabetic foot care.

**Conclusion:**

The review culminates in a prospective assessment of the trajectory of ML applications in the realm of diabetic foot care. We believe that despite challenges such as computational limitations and ethical considerations, ML remains at the forefront of revolutionizing treatment paradigms for the management of diabetic foot complications that are globally applicable and precision-oriented. This technological evolution heralds unprecedented possibilities for treatment and opportunities for enhancing patient care.

## 1 Introduction

Diabetes mellitus, a widespread global health issue, has emerged as the eighth leading cause of global disease burden (1). This condition affects hundreds of millions worldwide and is associated with several severe complications that pose a heavy load on global health systems. Notably, diabetic foot disease (DF), a common complication, affects approximately 18.6 million individuals annually, with a majority of cases progressing to foot ulcers, leading to amputation rates as high as 85% among those with diabetes (2–4). These dire outcomes not only impose immense physical and psychological tolls on patients but also strain healthcare systems financially. Notably, evidence-based interventions can effectively mitigate these consequences (5–7).

Since its initial identification, the DF has been recognized for its inherent complexity, posing a series of challenges that have captivated the academic community. One of the primary obstacles lies in the clinical heterogeneity among patients, which hampers the generalizability of research findings. This issue is further exacerbated by the limitations of existing diagnostic and therapeutic techniques, especially in the critical area of early diagnosis. Despite these challenges, the field has seen significant advancements, largely due to the integration of high-throughput genomics and advanced imaging technologies. These innovations have expanded the scope of DF research into an interdisciplinary endeavor, involving fields such as endocrinology, surgery, imaging, and bioinformatics (4). However, the current state of interdisciplinary collaboration falls short of the requisite level for tackling the multifaceted nature of the disease. Moreover, the influx of multimodal data from these diverse disciplines introduces additional complexities in data analysis (8). To navigate this intricate landscape, there is an increasing reliance on sophisticated analytical tools, such as machine learning(ML) and artificial intelligence, which hold the promise of transforming data integration and interpretation.

In recent decades, ML has evolved from a peripheral technology to a cornerstone in medical data analytics (9). Its transformative impact is evident across a broad spectrum of medical disciplines, ranging from oncology and cardiology to pulmonology (10–12). In clinical practice, ML techniques can mine key information from large amounts of medical data to provide doctors with more accurate diagnosis, prediction and treatment recommendations, and even provide decision support during surgery (9, 13). Notably, ML algorithms have proven to be invaluable in handling specialized data types, such as single-cell RNA sequencing medical imaging, and multi-omics data integration (14–16). In short, ML has revolutionized medical research and practice by autonomously “discovering” and optimizing algorithms to solve specific problems (17, 18).

Emerging evidence underscores the instrumental role of ML in advancing DF research. Utilizing ML algorithms, clinicians can now leverage biomarkers for the early diagnosis of DF, thereby initiating timely interventions (19–21). Furthermore, ML has been employed to predict the healing trajectory of DF ulcers, assess amputation risks, and formulate personalized treatment regimens (22, 23). Recent studies have even explored the use of ML in classifying thermal images of diabetic feet for early detection of complications (24, 25). Collectively, these applications not only deepen our understanding of the pathophysiological underpinnings of the DF but also herald new avenues for future research and treatment modalities.

This manuscript constitutes the first narrative review focusing on the applications of machine learning within the domain of DF pathology. In this narrative review, we systematically searched PubMed, Scopus, and Google Scholar up to September 2023, utilizing a detailed strategy with keywords focused on machine learning and diabetic foot pathology. Our approach, designed to capture the breadth and depth of the field, emphasizes the critical evaluation of machine learning principles and their application to diabetic foot care, supported by case studies and diagrams for clarity.

Central to this review are several pivotal contributions that advance the intersection of machine learning (ML) and diabetic foot (DF) pathology. Primarily, our comprehensive analysis elucidates the integral role of ML in enhancing diagnostic precision, prognostic accuracy, and the overall management of DF conditions. By synthesizing current research findings, we illustrate the substantial potential ML holds for revolutionizing patient care in this domain. Secondarily, we delve into the identification of emergent trends within this interdisciplinary sphere, spotlighting cutting-edge ML algorithms poised to significantly improve clinical outcomes. This exploration not only underscores the innovative strides being made but also serves as a beacon for future research directions, highlighting areas ripe for further exploration and technological advancement. Moreover, our candid discussion on the challenges and limitations inherent in the current landscape provides a balanced perspective, fostering realistic expectations among healthcare practitioners and researchers alike. In doing so, we aim to catalyze a more informed integration of ML technologies into clinical practice, bridging the gap between theoretical advancements and their practical application in improving diabetic foot care. Ultimately, the essence of our contribution lies in equipping healthcare professionals with a deep understanding of ML’s transformative potential, encouraging a collaborative, informed approach to leveraging these technologies for the betterment of DF management and patient outcomes.

## 2 Diabetic foot

### 2.1 Definition and pathophysiologic mechanisms of the diabetic foot

**Figure 1:**
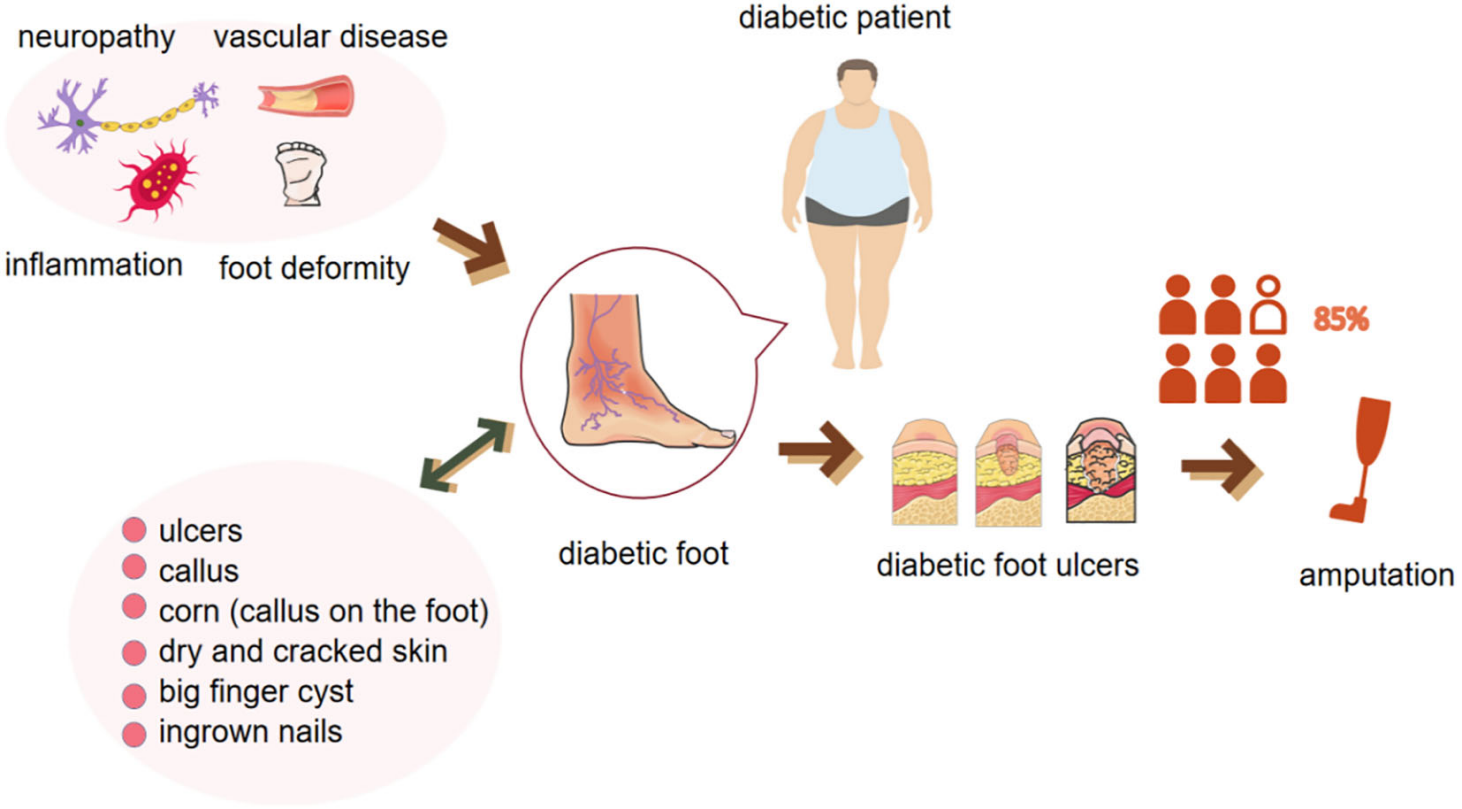
Pathologic factors, clinical manifestations of the diabetic foot and its further progression to severe ulceration and amputation.

The DF is a frequent and complex complication of diabetes mellitus, characterized by a multifaceted interplay of neuropathy, peripheral arterial occlusive disease, and infection (3) (**Figure 1**). The International Working Group on the Diabetic Foot formally defines the DF as a condition manifesting in patients with either a newly diagnosed or a historical presence of diabetes mellitus, featuring one or more of the following elements: peripheral neuropathy, peripheral arterial disease, infection, ulceration, neuro-osteoarthropathy, gangrene, or amputation (26).

The etiological factors contributing to the diabetic foot are often concomitant, exemplified by neuropathy, which is induced by a chronic hyperglycemic state and characterized by the impairment of sensory, autonomic, and motor nerves (2, 27, 28). This sensory dysfunction results in a diminished perception of pain, temperature, and pressure, thereby predisposing the foot to painless trauma and altered biomechanics (29). Concurrently, hyperglycemia-induced microangiopathy compromises blood circulation and leukocyte phagocytosis, thereby impairing wound healing and elevating the risk of infection (30). Moreover, peripheral arterial disease, a prevalent macrovascular complication in diabetes mellitus, is implicated in tissue loss in a significant subset of patients with diabetic foot ulcer (DFUs) (31). The foot serves as a complex target organ in the multisystemic pathology of diabetes. The convergence of these pathological processes culminates in a complex pathomechanism, rendering the foot particularly susceptible to ulceration and severe soft tissue infections (2). Therefore, the comprehensive management of diabetic foot necessitates an integrated treatment strategy, incorporating glycemic control, circulatory enhancement, infection prevention, and biomechanical optimization, with the overarching aim of mitigating the progression of diabetic foot and enhancing the quality of life for patients.

### 2.1 Diagnosis and treatment of the diabetic foot

The prevention and management of diabetic foot necessitate a collaborative, long-term approach involving the patient, physician, nursing staff, and other healthcare professionals. The International Working Group on the Diabetic Foot (IWGDF) has been instrumental in publishing and periodically updating evidence-based guidelines for the prevention and management of diabetic foot since 1999 (32–34). These guidelines underscore five pivotal domains: risk identification, routine foot examinations, structured patient and family education, footwear adaptation, and ulceration risk mitigation (7).

Initially, identifying the at-risk population is paramount. Annual screenings for diabetic patients with minimal ulceration risk are recommended to evaluate the loss of protective sensation and potential ulcer development (7). Subsequently, regular foot examinations facilitate early detection and intervention (2). In cases where high-risk factors are identified, comprehensive assessments are warranted, encompassing evaluations of nerve function, circulation, and musculoskeletal integrity (35). Recent advancements in radiology and nuclear medicine offer promising avenues for simplifying the evaluation of diabetic foot complications (36). Risk-stratification systems guide the frequency and scope of subsequent screenings (7), with high-risk patients requiring more frequent evaluations and specialist referrals (2).

Patient education serves as a cornerstone in diabetic foot prevention and is universally acknowledged for its role in halting disease progression (2). Patients must be educated about risk factors such as chronic hyperglycemia, smoking, and alcohol consumption, as well as the importance of routine foot care (37).Notably, a study conducted at the Diabetes Center in Khartoum, Sudan, revealed that only half of the participants had adequate knowledge and practice of self-foot care (37). Therefore it is urgent to improve patient’s continuous health education regarding diabetic foot self-care. For people with diabetic feet, all shoes should be adapted to any changes affecting the structure of the foot or the biomechanics of the foot (7). At the same time, comprehensive care for patients with diabetic foot disease should be provided, including recommendations for the preferred use of non-removable knee-height load-relieving devices, the use of removable knee-height or ankle-height load-relieving devices as a second choice for patients with contraindications, the use of offloading interventions to promote healing of foot ulcers and appropriate surgical treatment (34, 38).

For those diagnosed with diabetic foot, a multidisciplinary approach is imperative, encompassing preventive measures during hospitalization, therapeutic interventions, and seamless transitions from inpatient to outpatient care (39). Treatment objectives aim to achieve wound healing while preserving patient mobility (2). Innovations in pharmacotherapy, surgical interventions, and integrative treatment modalities have revolutionized diabetic foot management, ranging from basic pharmacological therapies and debridement to advanced techniques such as revascularization, decompression, and stem cell therapy (40–45). In the course of treatment, continuous monitoring by a multidisciplinary team is advised to adapt treatment strategies, ensuring both efficacy and safety (2).

## 3 Advancements in diabetic foot care: exploring machine learning applications in imaging diagnostics, biomarker detection, and clinical biomechanics

As summarized in many review studies, the application of ML in the general field of diabetes has been widely validated and recognized (46). However, compared to other diabetes-related complications such as diabetic retinopathy, relatively few studies have been conducted on diabetic foot syndrome (47). The complex pathophysiology underlying diabetic foot syndrome necessitates a transformative approach, where ML emerges as a pivotal tool, especially in the nuanced analysis of data derived from multiple sensors. Employing ML models offers a multi-faceted advantage in the medical field, significantly aiding physicians in accurately diagnosing diabetic foot, forecasting the potential clinical outcomes of diabetic foot ulcers, and issuing timely alerts regarding the imminent risks of amputation and mortality at the point of patient admission (48). Furthermore, these models play a crucial role in formulating targeted recommendations aimed at mitigating risk factors and enhancing the management and prevention strategies for diabetic foot ulcers. Given the burgeoning interest and ongoing research in this arena, the purpose of this manuscript is to provide a detailed review that not only underscores the current advancements but also illuminates the path for future investigative efforts in the realm of diabetic foot care.

### 3.1 Machine learning in diabetic foot diagnosis

#### 3.1.1 Application of machine learning to diabetic foot imaging diagnostics

Traditional wound assessment methodologies, often reliant on visual inspection, are subject to bias and contribute to clinical workload. In contrast, ML algorithms applied to image recognition facilitate a more nuanced and systematic evaluation of diabetic foot wounds, including their classification, localization, and dimensionality (49). ML has demonstrated considerable promise in the realm of imaging diagnostics for diabetic foot conditions. Notably, the integration of infrared foot thermography with ML algorithms has become increasingly prevalent in the evaluation and diagnosis of diabetic foot syndrome (24).

The infrared thermography is a fast, nonintrusive and non-contact method which allows the visualization of foot plantar temperature distribution (24). Previous research reports suggest that thermographic images could aid in detecting an increase in plantar temperature prior to the onset of DFUs However, the distribution of plantar temperature may be heterogeneous, complicating the quantification and use for predicting outcomes (25). Machine learning-based scoring techniques and feature selection have offered a robust solution for the early identification of diabetic foot. This model excels in optimizing features extracted from individual foot thermograms and surpasses the performance of deep learning methods reliant on 2D imagery. Such accuracy heralds the feasibility of deploying these models within smartphone applications, enabling patients to monitor DFUs progression autonomously within domestic settings (25). Further, unsupervised k-mean clustering of thermographic images to classify the severity of diabetic foot ulcers improves the accuracy and reliability of early and accurate diagnosis, enabling clinicians to proactively respond to the condition before it worsens. And, the use of advanced machine learning algorithms, including convolutional neural networks (CNNs) (e.g., the VGG 19 model), further improves the accuracy of diabetic foot severity detection and classification. This diagnostic accuracy is critical to the development of targeted treatment strategies, ensuring that patients receive the most appropriate interventions based on their specific risk profile (50).

The recent study on automated detection of lower extremity arterial stenosis using deep learning represents a significant leap forward in the clinical management of DF patients (51). By automating lower extremity arterial stenosis assessment, this method stands to streamline treatment planning significantly, overcoming the traditional reliance on time-consuming and inconsistent manual evaluations. The model’s utilization of 3D reconstructed blood vessel images and its adaptation of the YOLOv5 framework, enhanced with specific algorithmic modifications like the Convolutional Block Attention Module, achieves a remarkable mean Average Precision of 85.40% (51). This level of accuracy in identifying varying stenosis degrees directly translates into the potential for quicker, more precise identification of at-risk patients, facilitating the prompt initiation of personalized treatment strategies.

#### 3.1.2 Application of machine learning to diabetic foot biomarker diagnosis

The identification of biomarkers for diabetic foot conditions represents a pivotal frontier in early and precise diagnosis. ML algorithms, notably deep learning and Support Vector Machine (SVM), have been instrumental in the analysis and identification of potential biomarkers, thereby furnishing clinicians with invaluable insights into disease progression and therapeutic outcomes (20).

A investigation into the metabolomic changes associated with the progression from T2DM to DF has unveiled a set of predictive signatures specific to DF (52). This study highlights the transformative potential of metabolomic analyses in forecasting DF risk, revealing that differential metabolites in T2DM with DF patients, particularly those linked to branched-chain amino acid catabolic pathways, serve as distinctive markers for the disease’s progression (52). Employing advanced machine learning techniques, such as Lasso regression and random forest algorithms, the research offers new insights into the metabolic shifts characterizing the transition from T2DM to T2DM with DF. Moreover, a seminal investigation conducted by Wang et al., ML algorithms were employed to scrutinize genes associated with ferroptosis in the context of DFUs (21). The study culminated in the identification of 25 ferroptosis-related genes that could effectively discriminate between patients with DFUs and control subjects. Subsequently, a predictive model was successfully constructed utilizing ML algorithms.

Additionally, a recent study demonstrated the efficacy of deep learning models in utilizing coronary artery disease as an alternative biomarker for predicting cardiovascular disease and stroke risk in patients with diabetic foot infections (19). Collectively, these studies illuminate the transformative potential of ML in biomarker research for diabetic foot conditions, offering novel avenues for diagnosis and treatment.

#### 3.1.3 Machine learning in clinical biomechanics related to the diabetic foot

While it is well-acknowledged that diabetic foot conditions often manifest alterations in gait biomechanics, the application of ML in this domain remains conspicuously sparse.

It is noteworthy that Diabetic Peripheral Neuropathy is a primary precursor to diabetic foot ulcers and has always been the focus of innovative research efforts aimed at enhancing early detection and intervention. Recent studies, such as those utilizing Random Forest algorithms to analyze microcirculatory parameters like post occlusion reactive hyperemia, local thermal hyperemia, and transcutaneous oxygen pressure, have shown promising results in accurately diagnosing Diabetic Peripheral Neuropathy (53). These models have achieved significant accuracy, sensitivity, and specificity, highlighting the potential of ML to refine diagnostic criteria and more effectively predict patient outcomes compared to traditional methods. By leveraging ML models, healthcare professionals can now more accurately identify individuals at risk, thereby enabling timely and targeted interventions.

As a matter of fact, the presence of gait specificity among patients poses significant challenges to traditional analyses of electromyography (EMG) and ground reaction forces (GRF) in diagnosing Diabetic Neuropathy (DN) and DFUs (54). Individual differences, including age, gender, body composition, disease status, and history of injury, contribute to unique gait patterns, leading to considerable variability even within the same experimental groups. This variability is particularly pronounced in DN and DFUs conditions, where sensory loss, muscle weakness, and structural alterations in the lower limbs result in adaptive gait changes aimed at minimizing pain or discomfort. Such adaptations can vary widely across individuals, complicating the use of standard EMG and GRF analyses to reliably identify biomechanical changes associated with DN and DFUs (54). These challenges highlight the critical significance of integrating machine learning techniques, renowned for their adeptness in managing highly individualized data and discerning intricate patterns. Such capabilities are instrumental in enabling early interventions and crafting customized treatment strategies, thereby enhancing patient care and outcomes.

### 3.2 Application of machine learning in prognostic assessment of diabetic foot

#### 3.2.1 Application of machine learning in prediction of diabetic foot ulcer healing

Machine learning serves as a pivotal instrument in prognosticating the healing course of DFUs. The advent of smartphones and smart tools has further simplified this measurement process (55, 56). A prospective cohort investigation substantiated that elementary wound characteristics, such as wound surface area and duration, could be employed via machine learning algorithms to reliably forecast wound resolution by the 16th week of clinical management (23). In a seminal study by Renaid et al., electronic health records from 2,291 visits involving 381 ulcers across 155 patients were utilized to construct a ML model. This model employed both clinical attributes and image features to prognosticate the healing trajectory of diabetes-related foot ulcers (57). The findings underscore the model’s potential for remote prognostic assessment via smartphone-captured imagery (57). The study also found that crucial predictive features predominantly included hand-crafted imaging attributes, while clinical factors like nutritional status and ulcer size were less frequent yet impactful, underscoring their known influence on wound healing (57).

Machine learning models showcased higher predictive accuracy compared to traditional clinical or molecular biomarker-based models, affirming their potential in DFUs care. These findings have significant implications for telemedicine, suggesting that smartphone or tablet images could effectively monitor DFUs healing, offering a practical, non-invasive method easily integrated into clinical practice. The efficiency of hand-crafted image features in these models also means that their deployment does not require extensive computational resources, facilitating broader clinical adoption. And a study highlights the clinical relevance of adopting sophisticated machine learning models in DFUs management, emphasizing the integration of RGB images with texture data to enhance diagnostic accuracy. Such advancements promise to streamline early DFUs detection, alleviate the clinical workload through diagnostic automation, and reduce misdiagnosis risks (58). Furthermore, the demonstrated ability to classify DFUs by severity and type paves the way for more personalized treatment approaches, enabling clinicians to devise targeted interventions that cater to the unique needs of each patient, thus optimizing DFUs treatment outcomes (58).

A noteworthy study has introduced an advanced prediction model that melds clinical insights with genetic data to forecast DFUs healing outcomes more accurately (59). This innovative approach, involving a cohort study of 206 patients, incorporates not only clinical factors and circulating endothelial precursor cell measurements but also delves into the genetic realm by examining the NOS1AP gene’s single nucleotide polymorphisms (59). By employing a blend of statistical and machine learning techniques, the study developed prognostic models that significantly surpass traditional clinical models in predictive accuracy. Moreover, it provides a solid foundation for clinical trial designers to identify candidates more likely to benefit from new therapies.

Using machine learning and genomics, the study of DFUs uses the comprehensive database of Gene Expression Omnibus (GEO) database to analyze the complex pathological mechanism behind the disease, including vascular changes, neuropathy and infection (60). A notable study analyzed microarray data from the GEO database using advanced bioinformatics and ML techniques, such as LASSO and SVM-RFE, to spotlight GSTM5 as a crucial immune-related biomarker for DFU (61). This discovery, validated through external datasets and immunohistochemistry, underscores GSTM5’s potential influence on essential signaling pathways and its association with T cells, paving the way for targeted immune therapies (61). Equally important is that another pivotal study sheds light on glutamine metabolism’s role in DFU pathogenesis (62). By analyzing microarray datasets from the GEO database, this research unveiled differential expressions of glutamine metabolism-related genes and their correlation with immune cell infiltration in DFU patients. The employment of a SVM model, based on 5 critical genes, demonstrated remarkable predictive accuracy (AUC = 0.929) on external validation datasets (62). Furthermore, investigations into angiogenesis-related genes using machine learning algorithms underscored the vital function of angiogenesis in DFU’s wound healing process (63). Merging data from several datasets led to the identification of thrombomodulin as a critical gene influencing angiogenesis during DFUs development, validated by external datasets and biological assays. These findings collectively advocate for the application of machine learning in uncovering novel DFUs biomarkers, providing invaluable insights into the disease’s molecular biology, and paving the way for personalized therapeutic strategies.

#### 3.2.2 Application of machine learning to diabetic foot amputation and mortality risk assessment

DFUs are acknowledged as one of the most grievous sequelae of diabetes, markedly elevating the amputation risk profile in affected individuals. The use of image-centric machine learning algorithms, especially convolutional neural networks, has advanced the early detection of DFUs to prevent limb amputation and infections (22, 58). These algorithms assess critical parameters, including wound infection, offering benefits not only in early DFUs detection but also in reducing clinical workloads, enhancing cost-effectiveness, standardizing treatment, improving patient care, and minimizing the incidence of misdiagnoses (58). Machine learning significantly augments clinical decision-making in the management of DFUs by pinpointing critical factors that influence the likelihood of amputation. Consequently, this fosters an environment where interventions are not only timely but also tailored to the specific risk profiles of individual patients, thereby optimizing outcomes in the management of DFUs.

To refine amputation risk assessments during hospitalization for DFUs patients, a study scrutinized 618 DFUs inpatients, segregating them into non-amputation, minor amputation, and major amputation cohorts. The machine learning model devised in this study not only accurately gauged the amputation risk but also furnished invaluable insights for personalized risk stratification (64). Further, the study employs Extreme Gradient Boosting (XGBoost) and Gradient Boosted Trees algorithms to achieve remarkable predictive accuracies for each category of LEA risk, highlighted by ROC scores of 0.820 for major LEA events, 0.637 for minor LEA events, and 0.756 for any LEA event (65). A critical aspect of this study is its utilization of SHapley Additive exPlanations (SHAP) for model interpretability, a feature that distinguishes this research by providing clear insights into the contributing factors behind the model’s predictions. Key determinants such as total white cell count, comorbidity score, red blood cell count, eosinophil levels, and the presence of necrotic eschar in wounds were identified as significant predictors of LEA risk. This model’s ability to not only accurately predict LEA risk but also offer transparent, explainable insights into the underlying factors marks a significant advancement in DFUs care.

In a comprehensive study encompassing 326,853 DFUs -related hospital admissions, machine learning algorithms accurately predicted the likelihood of major lower extremity amputations with a precision of 77.8% and an AUC of 0.84 using five clinical variables: gangrene, osteomyelitis, peripheral vascular disease, systemic infection, and weight loss (66). Notably, gangrene emerged as the most critical risk factor, substantially increasing amputation risk. This aligns with meta-analyses identifying gangrene as a key predictor, emphasizing infections as major risk factors for amputation. The study also reveals the prognostic value of weight loss, underscoring it as a modifiable factor that clinicians can target to slow disease progression.

A considerable subset of patients with DFUs necessitate minor amputations. Early discernment of such outcomes is instrumental in guiding clinical decision-making and mitigating the incidence of major amputations and mortality (67). More importantly, the application of machine learning and feature significance analysis was utilized to identify and assess which factors were most critical in predicting the likelihood of minor amputation. For instance, the study finds that random blood glucose levels, history of DFUs, and serum albumin are the most important factors in predicting the need for minor amputations (67). Optimal blood glucose control is paramount, aligning with the ACCORD trial’s findings that tight glycemic management significantly reduces amputation risks. A history of DFUs underscores the necessity for diligent monitoring to prevent recurrence, while adequate serum albumin levels, indicative of nutritional status, are crucial for effective wound healing. This analysis directs clinicians towards targeted interventions in glycemic stability, nutritional support, and comprehensive follow-up, showcasing machine learning’s role in advancing DFUs patient care (67).

Moreover, Du et al. ascertained that the XGBoost model could furnish evidence-based risk profiles concerning amputation and mortality during the COVID-19 pandemic, thereby benefiting DFUs patients (48). In the study examining amputation and mortality among DFUs patients during the COVID-19 pandemic, machine learning techniques were instrumental in identifying key clinical variables influencing outcomes (48). By analyzing patient data, the machine learning model pinpointed white blood cells, blood potassium, and prehospital status as pivotal predictors for amputation before lockdown, and prehospital status, foot ischemia, and serum albumin post-lockdown (48). For mortality, critical variables included nonfoot infection, age, and foot infection. The subsequent analysis by another study extends this narrative by employing machine learning to prognosticate long-term mortality risks in DFUs patients, leveraging multilayer perceptron classifiers for 5-year and 10-year mortality predictions (68). This approach, rooted in the analysis of comprehensive clinical predictors, reinforces the critical role of infections and underlying health conditions in DFUs patient outcomes (68). Notably, the study’s ability to predict mortality with notable accuracy using non-invasive and economical predictors further emphasizes ML’s potential to refine risk assessments and guide clinical decision-making.

Schäfer et al. utilized existing socioeconomic registries and clinical records from 246,705 diabetic patients to compile data on socioeconomic status, medical history, and key risk factors (69). By using machine learning to analyze these extensive clinical databases, research has found that patients with lower household incomes face higher risks (69). Their research goes beyond traditional medical and physical parameters, emphasizing the significant impact of socio-economic and demographic factors on DFUs outcomes. This insight underscores the importance of integrating medical care with socio-economic support, aiming not only to mitigate the physical risks associated with DFUs but also to address the underlying socio-economic barriers that exacerbate health disparities among diabetic patients. Ultimately, this guidance steers clinicians towards more effective and equitable DFUs management practices.

## 4 Comparative analysis of machine learning algorithms: evaluating optimal model performance in diabetic foot clinical studies

In the realm of diabetic foot pathology, various machine learning methodologies manifest distinct capabilities across a spectrum of tasks, each tailored to its unique features and functionalities. These computational tools furnish researchers and clinicians with an expansive arsenal for the nuanced understanding, prediction, and therapeutic management of this intricate medical condition. A compendium of prevalent machine learning techniques and their respective applications in the domain of diabetic foot care is delineated in **Table 1**:

**Table 1:** Different application functions of different machine learning methods in diabetic foot research.

Category	Subcategory	Algorithms	Specific Features	Literature applications
Supervised learning	regression (statistics)	linear regression	Simplicity and interpretability	(54)
	categorization	Decision Trees and Random Forests	Interpretability; Feature Selection: Help researchers identify key biomarkers or risk factors.	(48, 57, 66, 67)
		support vector machine	Classification tasks: Such as distinguishing diabetic foot from other foot disorders.	(48, 57, 67, 70)
		logistic regression	Used to predict the risk of diabetic foot, e.g., based on the patient’s age, gender, and history of diabetes.	(48, 67)
	Deep Learning and Neural Networks	Convolutional Neural Network	Used to analyze medical images of the foot, such as X-ray, MRI or ultrasound images, to detect early signs or complications of diabetic foot.	(25, 48, 50, 58).
unsupervised learning	clustering	K-Means	subgroup analysis	(50).
transfer learning			Enhancing model performance using pre-trained models from related fields, e.g., diabetic retinopathy, when diabetic foot data is limited	(49).
Integrated Methods		Boosting	Stepwise optimization: an advantage when dealing with diabetic foot data with complex features and nonlinear relationships.	(25, 48, 50, 64, 67)

Simple linear models (i.e., logistic regression model, Cox proportional hazard model), which were developed to evaluate the risk of amputation in patients with DFUs, were limited by the fact that they only predict a single outcome (the possibility of amputation) and cannot distinguish between minor and major amputation. Whereas these models can incorporate more risk factors, they are limited in capturing the non‐linear relationship among the risk factors, and are prone to underfitting, leading to low accuracy and stability. The emergence and development of machine learning algorithms provide new opportunities to overcome the challenges presented by the simple linear models.

The clinical importance of comparing machine learning research models in the context of diabetic foot management lies in the ability to discern which methodologies most effectively predict, diagnose, and stratify risk levels among patients. In this section, we focus on evaluating the application of machine learning technologies in the prediction and detection of diabetic foot disease through quantitative research methods. Specifically, we conduct a detailed comparative analysis of the optimal model performance across a series of key studies. These analyses are based not only on metrics, numerical results, and statistical significance but also include direct comparisons with other machine learning algorithms. Such comparative analyses enable healthcare professionals to select the most accurate and reliable tools, ensuring that interventions are both timely and tailored to individual patient needs. This not only enhances patient outcomes by facilitating early detection and personalized treatment plans but also contributes to the optimization of healthcare resources, reducing both the clinical burden and the overall incidence of severe complications associated with diabetic foot ulcers (**Table 2**).

**Table 2:** Comparison of optimal model performance in clinical studies.

Name of study	Optimal Model	AUROC	Accuracy	Precision	Recall	F 1 Score
The amputation and mortality of inpatients with diabetic foot ulceration in the COVID-19 pandemic and post pandemic era: A machine learning study (48)	XGBoost	0.86 (Amputation), 0.94 (Mortality)	80 (Amputation), 90 (Mortality)	Not Provided	67 (Amputation), 100 (Mortality)	Not Provided
Image segmentation using transfer learning and Fast R-CNN for diabetic foot wound treatments (50).	VGG 19 CNN	Not Provided	95.08	95.08	95.09	95.08
Machine Learning-Based Diabetic Neuropathy and Previous Foot Ulceration Patients Detection Using Electromyography and Ground Reaction Forces during Gait (54)	KNN (Optimized)	0.99	95.80	95.86	95.80	95.78
Utilization of smartphone and tablet camera photographs to predict healing of diabetes-related foot ulcers (57)	SVM	0.734	81.1	82.8	92.3	87.3
An explainable machine learning model for predicting in-hospital amputation rate of patients with diabetic foot ulcer (64)	multi-class classification model	0.90	Not Provided	86.3	87.1	Not Provided
A Machine Learning Model for Prediction of Amputation in Diabetics (66)	boosting	0.84	77.8	Not Provided	76.1	Not Provided
Machine learning for the prediction of minor amputation in University of Texas grade 3 diabetic foot ulcers (67)	XGBoost	0.881	0.811	0.828	0.923	0.873
Area Determination of Diabetic Foot Ulcer Images Using a Cascaded Two-Stage SVM-Based Classification (70)	SVM	Not Provided	Not Provided	Not Provided	71.4(entire image)- 74.5 (wound + healthy skin only)	Not Provided

Study Title: A brief description of the study's theme and objectives.

Optimal Model: The machine learning model that performed best in the study.

AUROC: A metric of model performance, with higher values indicating better classification capabilities of the model.

Accuracy: The proportion of correct predictions made by the model.

Precision: The ratio of true positive predictions to the total number of positive predictions made.

Recall: The ratio of true positive predictions to the total number of actual positives.

F1 Score: The harmonic mean of precision and recall, used to measure the model's accuracy.

Within the realm of DFUs diagnostics, two pivotal studies have illuminated the potential of ML and CNNs to refine classification and severity grading methodologies. The first investigation harnesses unsupervised ML techniques, notably K-means clustering, to recalibrate the classification within a publicly accessible heat-spectrum dataset, aimed at discerning diabetes severity levels (50). This methodological innovation yielded impressive outcomes, with the model demonstrating an accuracy, precision, sensitivity, and F1 score of 95.08%, complemented by a specificity of 97.2% in the stratification of severity. Such findings validate the nuanced capacity of ML in analyzing complex thermal imagery associated with DFUs, thereby enhancing the granularity of severity grading. Building upon this foundation, a subsequent study further explores the capabilities of ML in DFUs management, with a specific focus on the application of CNNs (58). This research ventures beyond conventional RGB imagery, incorporating diverse data inputs to bolster CNN model performance in DFUs classification. By integrating texture information, derived via the mapping binary pattern technique, with standard RGB images, the study posits a nuanced approach that markedly augments DFUs detection accuracy. This methodology unfolds in a two-stage process: initially extracting texture data from RGB images, subsequently leveraging this information to fortify the CNN’s proficiency in DFUs identification. Such integration of texture features with RGB data as fused image inputs for CNNs underscores the significance of multidimensional data analysis in achieving enhanced classification outcomes, thus promising improved diagnostic accuracy and, consequently, patient prognosis.

A study investigated the use of EMG and GRF parameters to identify patients with DN and DFUs through the k-nearest neighbors (KNN) algorithm. In comparison with seven other commonly used machine learning algorithms (including discriminant analysis, kernel models, linear classification, etc.), the optimized KNN model particularly excelled in the EMG analysis of the GL and VL muscle combination, achieving an impressive accuracy of 95.80% and an AUC value of 0.99. These results highlight the potential and advantage of KNN in processing specific biomechanical data (54).

In the quest to advance the prognosis and treatment of DFUs, a pivotal study has leveraged the SVM algorithm to forecast the healing trajectories of these complex wounds (57). Demonstrating commendable efficacy, the SVM algorithm outshone traditional models such as logistic regression and Naive Bayes on a validation set, manifesting a robust performance characterized by an Accuracy of 0.811, Precision of 0.828, Recall of 0.923, and an F1 score of 0.873 (57). Notably, when comparing an SVM_test model, which utilized solely hand-crafted image features, to one incorporating a full spectrum of features, it became evident that the former’s higher AUROC value of 0.794, against the latter’s 0.734, did not eclipse the holistic advantage of utilizing all available features (57). This comprehensive approach underscores the model’s adeptness in achieving a balanced precision-recall trade-off and its exceptional capacity in accurately pinpointing true positive DFUs cases, thereby enhancing diagnostic precision. Bridging this analytical prowess to the broader spectrum of DFUs management, it becomes apparent that the incorporation of hand-crafted imaging features—encompassing both color and texture information—significantly amplifies the predictive accuracy of machine learning models beyond what is achievable with deep learning or clinical data alone (57). This innovative strategy underscores the critical role of nuanced, hand-crafted features in enriching the machine learning landscape for DFUs diagnosis and management. Such features, particularly evident in the analysis of mean green color intensity, offer novel clinical insights by highlighting the importance of non-red hues in ulcers, which may signal the presence of severe infections. This methodological insight not only showcases the superior performance of models trained on hand-crafted features but also heralds a new era of precision in DFUs management, where simple yet powerful imaging attributes can guide more nuanced and effective clinical interventions.

The application of Light Gradient Boosting Machine, a state-of-the-art machine learning algorithm known for its efficiency and effectiveness in handling large datasets, stands at the core of this predictive endeavor (64). Coupled with the SHAP, the model transcends mere prediction, offering insights into the interpretability of its prognostic assessments. The achieved AUC values of 0.90, 0.85, and 0.86 for predicting non-amputation, minor amputation, and major amputation outcomes, respectively, underscore the model’s robust predictive capability (64). This fusion of Light Gradient Boosting Machine and SHAP not only enhances the accuracy of amputation rate estimations during hospitalization but also pioneers a path toward individualized risk factor analysis among DFUs patients.

XGBoost is an ensemble learning algorithm that combines the predictions of multiple trees and sums their prediction scores to obtain a final score. With advantages in speed, efficiency, and fault tolerance, XGBoost can effectively avoid overfitting and demonstrates a high generalization capability. The performance of the XGBoost algorithm surpasses that of commonly used linear regression models (LR), single classifiers (DT and SVM), and another ensemble learning machine learning algorithm (RF). Many researchers have proven that XGBoost performs well in predicting a variety of clinical diseases. In a study encompassing 362 cases of grade 3 diabetic foot ulcers, a variety of machine learning algorithms, including Decision Trees, Random Forest, Logistic Regression, SVM, and XGBoost, were used to independently construct risk prediction models (67). Compared to other machine learning algorithms, the XGBoost algorithm demonstrated superior performance in predicting whether patients with diabetic foot ulcers would require minor amputation surgery, with an AUROC value of 0.881, an accuracy of 81.1%, and an F1 score of 87.3% (67). These results highlight the efficiency and accuracy of XGBoost in processing complex medical data.

One innovative approach employs SVM to delineate wound boundaries in foot ulcer images, captured via specialized imaging apparatus (70). This research marks a significant innovation in DFUs management by introducing a smartphone-based system that employs machine learning for precise wound boundary determination. Utilizing a sophisticated cascaded two-stage SVM classifier, this system analyzes high-resolution images to accurately identify wound regions, leveraging both color and texture descriptors. The involvement of clinicians in generating accurate training labels ensures the model’s reliability and effectiveness. Demonstrating superior sensitivity compared to traditional machine learning approaches, this system offers rapid wound assessment directly from a smartphone, significantly enhancing the practicality of DFUs care (70).

Synthesizing these studies, we are afforded not only a view of the unique contributions each model makes to the prediction and detection of diabetic foot disease but also insights into the relative efficacy of different algorithms when processing specific types of data, based on comparisons between models. These comparisons offer valuable perspectives for future research, particularly in the selection and optimization of machine learning models to develop highly accurate clinical predictive tools.

## 5 Datasets

The exploration of ML applications for the early detection of DF highlights the critical importance of leveraging diverse datasets. In the realm of DFU research, datasets such as STANDUP, INAOE, and local collections provide invaluable insights into the complex thermal patterns associated with diabetic foot pathology (71). These datasets, characterized by variations in infrared image acquisition parameters, population samples, and health conditions, serve as foundational elements for developing and validating predictive models.

### 5.1 INAOE dataset

The advances in infrared thermography during recent years have opened new possibilities for its use in medical diagnosis. The detection of complications related to diabetic foot is one of the many uses of this technology. A new public plantar thermogram database composed of 334 plantar thermograms from 122 diabetic subjects and 45 non-diabetic subjects (72). Each thermogram includes four extra images with their respective temperature file, corresponding to the four plantar angiosomes. The database is expected to provide a valuable source to promote research about the potential of infrared thermography for the early diagnosis of diabetic foot problems. Further, The INAOE dataset has been enhanced by the inclusion of a local dataset acquired in 2021, which comprises images from 22 healthy volunteers recorded at four distinct time points, specifically utilizing those captured after a 15-minute resting period (71, 73). This local dataset was merged with the INAOE dataset to create an extended collection aimed at balancing the previously noted skew towards diabetic cases. This strategic amalgamation enriches the dataset’s diversity, offering a more robust foundation for DF detection and prevention research.

### 5.2 STANDUP dataset

This research database comprises 415 multispectral images (thermal and RGB) of the plantar foot, with 125 images from healthy individuals and 290 from type II diabetic patients (74). Healthy participants were from PRISME, France, and IRF-SIC, Morocco, while diabetic subjects were recruited from Hospital Nacional Dos de Mayo, Lima, Peru, for a study on diabetic foot ulcer detection. The database aims to support research in early ulcer detection by providing details on recruitment, acquisition protocols, and equipment used, facilitating the creation of similar databases for advancing diabetic foot research.

### 5.3 Zivot dataset

The Zivot dataset marks a significant advancement in DFUs research, addressing the need for comprehensive data to overcome previous limitations of disparate and inadequate datasets (75). This dataset incorporates a broad spectrum of data, including red–green–blue images, temperature, moisture, and patient demographics, enabling a nuanced evaluation of DFUs. Developed in collaboration with the Zivot Limb Preservation Centre, it introduces a meticulously designed data collection protocol intended as a benchmark for DFUs research. The inclusion of advanced imaging tools like depth cameras, red–green–blue sensors, and thermometry enhances the precision of DFUs diagnoses. The application of machine learning and deep learning on the Zivot dataset demonstrates promising accuracy, as shown by F1-score and mAP segmentation metrics, suggesting the dataset’s potential to drive forward holistic and multimodal DFUs research approaches.

### 5.4 The National Inpatient Sample database

The National Inpatient Sample (NIS) from the Healthcare Cost and Utilization Project adds a pivotal dimension to DFUs research (66). Covering admissions for DFUs diagnosis from 2008 to 2014, the NIS dataset provides detailed insights into patient demographics, comorbidities, and outcomes through ICD-9-CM codes and AHRQ measures (66). Focusing on major lower extremity amputation cases, the NIS’s comprehensive approach enriches DFUs research with a broad patient base, involving over 326,853 cases after meticulous data cleaning. Through advanced statistical and machine learning analysis, the dataset aids in developing predictive models for amputation risks, utilizing decision trees and Lasso regression among other methods.

### 5.5 Augmented DFU classification datasets

Recent advancements introduced the Part-A and Part-B datasets, enriching machine learning model training for DFUs research through data augmentation (58). The Part-A dataset, initially comprising 1,679 images, expanded tenfold to 16,790 images, utilizing rotation, flipping, and color space modifications (58). The Part-B dataset focuses on ischemia and infection, offering detailed classification with augmented patches for precise model training (58). A 10-fold cross-validation strategy over five iterations ensures exhaustive model evaluation, employing training, validation, and testing sets to optimize accuracy and reliability. These augmented datasets exemplify how strategic data enhancement and validation methodologies are crucial for advancing DFU diagnosis and management, highlighting machine learning’s transformative potential in medical research.

### 5.6 Gene expression omnibus database

The GEO database, maintained by the National Center for Biotechnology Information, is a public repository that archives and freely distributes comprehensive sets of microarray, next-generation sequencing, and other forms of high-throughput functional genomic data. Established to support the research community’s efforts in molecular biology, GEO serves as a critical resource for the study of gene expression across a vast array of conditions, diseases, and experimental treatments. GEO allows researchers to submit, retrieve, and explore data sets in an effort to understand complex biological phenomena. This includes the examination of gene expression changes associated with diseases such as DFUs, where understanding the genetic underpinnings can lead to breakthroughs in diagnosis, treatment, and prevention strategies (61–63). The database supports the use of machine learning techniques to analyze and interpret the vast amounts of genomic data it contains, enabling the identification of disease biomarkers and the elucidation of molecular pathways involved in the progression of complex conditions like DFUs (61–63).

The juxtaposition of these datasets reveals the inherent challenges in dataset imbalance, where diabetic cases often outnumber healthy controls, and the difficulty in standardizing feature extraction methodologies. Such disparities necessitate meticulous dataset selection and preprocessing strategies to ensure model robustness and generalizability. Moreover, the introduction of datasets like STANDUP enriches the field by offering an expanded pool of samples, thereby enhancing the potential for ML and DL models to accurately generalize across varied patient demographics.

This diversity among datasets underscores the necessity for continuous evaluation and refinement of ML and DL methodologies, ensuring that the models developed are not only precise but also applicable across different clinical settings. Consequently, the selection and critical review of datasets emerge as pivotal steps in the advancement of DFU detection technologies, driving forward the pursuit of more effective, personalized patient care.

## 6 Results

The realm of diabetic foot care is witnessing a transformative era, marked by the integration of ML into its diagnostic, prognostic, and therapeutic strategies. The diabetic foot, a complex interplay of neuropathy, infection, and ischemia, poses significant challenges in its management, necessitating advanced approaches for early detection, precise diagnosis, and effective treatment. Innovations in ML applications to diabetic foot imaging diagnostics have revolutionized the ability to identify subtle changes in foot health, offering a leap forward from traditional visual inspections to nuanced, systematic evaluations. Through the utilization of algorithms adept at analyzing images for classification, localization, and dimensionality, ML facilitates a comprehensive understanding of DFUs, enabling early intervention and personalized care. Equally critical is the application of ML in the identification of biomarkers for diabetic foot conditions. The exploration of genomic data and the analysis of potential biomarkers through ML algorithms furnish clinicians with insights into disease progression and therapeutic outcomes, enhancing the precision of diagnoses and the tailoring of treatment plans. Clinical biomechanics related to the diabetic foot also benefit from ML’s prowess. The assessment of gait abnormalities, pressure distribution, and other biomechanical factors through ML algorithms aids in the early detection of at-risk individuals, guiding interventions to prevent ulcer formation and progression. The prognostic assessment of diabetic foot through ML, particularly in predicting DFU healing and assessing amputation risk, underscores the utility of ML models in clinical decision-making. These models identify critical factors influencing DFU outcomes, enabling timely and customized interventions to mitigate the risk of severe complications. The comparative analysis of ML algorithms, as presented in our review, showcases the diverse approaches and their efficacy in diabetic foot clinical studies. By evaluating the optimal model performance, healthcare professionals are equipped with evidence-based tools for improving patient care in diabetic foot management. Lastly, the role of datasets in advancing ML applications cannot be overstated. The review highlights key datasets such as STANDUP, INAOE, and Zivot, among others, that provide the foundational data necessary for developing and validating predictive models. These datasets, with their vast and varied information, are crucial in fostering innovations that drive forward the application of ML in diabetic foot care. Collectively, these sections of the review articulate the significant impact of ML on the management of diabetic foot, from diagnostics to treatment strategies. The integration of ML not only augments clinical decision-making but also aligns with the objectives of precision medicine, heralding a new chapter in the fight against diabetic foot complications.

## 7 Discussion and outlook

### 7.1 Discussion of the findings

While extant literature has acknowledged the utility of machine learning in the broader context of diabetes management (76), a focused examination of its applicability to diabetic foot conditions remains conspicuously absent. A recent review did venture into artificial intelligence-based investigations of the diabetic foot but offered only cursory treatment of machine learning methodologies (77). It merits clarification that artificial intelligence serves as an overarching discipline, within which machine learning operates as a specialized subset, primarily concerned with algorithmic and statistical learning from data. In medical contexts, machine learning excels in the manipulation of extensive data sets, such as genomic sequences and medical imaging, and demonstrates proficiency in disease prediction and diagnosis (9, 10, 78). To the best of our scholarly awareness, this constitutes the inaugural machine learning-centric review explicitly addressing diabetic foot conditions.

### 7.2 Future trajectories of machine learning in diabetic foot research

#### 7.2.1 Tailored therapeutic approaches

Machine learning has exhibited exemplary efficacy in the early diagnosis, risk stratification, and prognostic evaluation of diabetic foot conditions, thereby holding substantial promise for its integration into personalized treatment paradigms. This encompasses, but is not confined to, the customization of pharmacotherapy, optimization of therapeutic regimens, and telemedical monitoring. Through granular analysis of expansive patient data, machine learning algorithms can furnish clinicians with highly individualized treatment recommendations, thereby enhancing therapeutic outcomes, mitigating complication risks, and elevating patient satisfaction and quality of life (79).

#### 7.2.2 Interdisciplinary synergies

As computer science and IoT technologies continue to evolve, we foresee a deeper intersection and collaboration between ML and bioinformatics, clinical medicine, and other related disciplines. This collaboration will not only be limited to current areas of widespread interest, such as advanced medical image analysis and early diagnostic techniques, but will also extend to emerging technology areas, such as wearable medical devices. These devices, combined with ML capabilities, are expected to provide real-time remote monitoring and management of diabetic foot patients, resulting in a more efficient and convenient healthcare experience for them. In addition, this interdisciplinary collaboration will further advance the application of ML technology in diabetic foot treatment, making it more precise and personalized to better meet patients’ treatment needs.

#### 7.2.3 Global health implications

Diabetic foot complications have ascended to the forefront of global public health challenges, particularly in low- to middle-income nations. In confronting this exigency, avant-garde technologies like machine learning, especially in the realms of transfer learning and federated learning, offer a robust framework for the delivery of efficient and high-caliber healthcare in resource-constrained settings. For instance, the application of pre-trained algorithmic models enables medical specialists to render precise diagnostic and therapeutic recommendations in the absence of extensive training data sets. More critically, machine learning can facilitate the judicious allocation of essential medical resources, thereby optimizing the global response to this burgeoning public health crisis.

## 8 Challenges and possible solutions to the application of machine learning in the diabetic foot

While ML has made considerable strides in the prognostication and therapeutic management of diabetic foot conditions, several formidable challenges persist. These include, but are not limited to, issues surrounding model interpretability, data quality, and data imbalance, which collectively constitute the crux of ongoing research endeavors (80).

### 8.1 Data quality and imbalance

The performance of a ML model depends heavily on the quality and quantity of the input data. In diabetic foot studies, data may be missing, inconsistent, or excessively noisy. In addition, there may be far more healthy samples than diabetic foot samples, leading to bias in model training.

### 8.2 Maintaining privacy

The data-intensive nature of machine learning amplifies the imperative for stringent safeguards surrounding patient data privacy. Despite researchers’ efforts to minimize data privacy risks, completely eliminating them remains a challenge.

### 8.3 Model interpretability

The enigmatic “black box” decision-making paradigm inherent to ML poses a significant challenge. Clinicians require transparent decision logic to make judicious clinical determinations. The complexity of certain algorithms may obfuscate error recognition or bias identification, thereby undermining patient trust (81, 82). To surmount these challenges, potential strategies include:

(1) Employing advanced data preprocessing techniques to optimize data quality, coupled with data augmentation methods to diversify the sample pool;
(2) Implementing oversampling or under sampling strategies to rectify data imbalances and engender more equitable models;
(3) Utilizing interpretability tools such as LIME or SHAP to elucidate model decision-making processes, thereby bolstering model credibility in clinical applications;
(4) Incorporating differential privacy and federated learning methodologies to fortify data security and privacy;
(5) Fostering interdisciplinary dialogue and collaboration between ML and medical experts to facilitate mutual understanding;
(6) Continual validation and scrutiny of ML models within authentic clinical environments to ascertain their precision and reliability;
(7) Assembling a multidisciplinary consortium comprising specialists from diverse fields—ranging from diabetology and various surgical disciplines to infectious disease experts and podiatrists—to deliver comprehensive patient care (4). If immediate formation of a full-fledged team is impractical, a phased approach to team assembly should be pursued, incorporating as many specialties as feasible.

In an era marked by rapid technological advancements and burgeoning interdisciplinary collaborations, the application of machine learning in the realm of diabetic foot care holds unparalleled promise and potential. We are both hopeful and confident that as research in this domain deepens, scholars and clinicians alike will continue to propel the innovation and implementation of these advanced computational techniques. Such endeavors aim to furnish more precise and efficacious therapeutic strategies for patients afflicted with diabetic foot complications, thereby making a seminal contribution to the global healthcare landscape.

## Author contributions

HG: Writing – original draft. YW: Writing – original draft. PN: Writing – original draft. YuZ: Writing – original draft. YaZ: Writing – original draft. RM: Writing – original draft. XF: Writing – original draft. RY: Writing – original draft. SZ: Writing – original draft. JL: Conceptualization, Writing – review & editing. JT: Writing – original draft.
