# Supplementary material for: The role of machine learning in advancing diabetic foot: a review
Source: Front Endocrinol (Lausanne). 2024 Apr 29;15:1325434. doi: 10.3389/fendo.2024.1325434 (PMC11089132; doi:10.3389/fendo.2024.1325434)
Supplement: Supplementary file 1 [file DataSheet_1.docx]

***Supplementary Material***

**Figures and Tables**


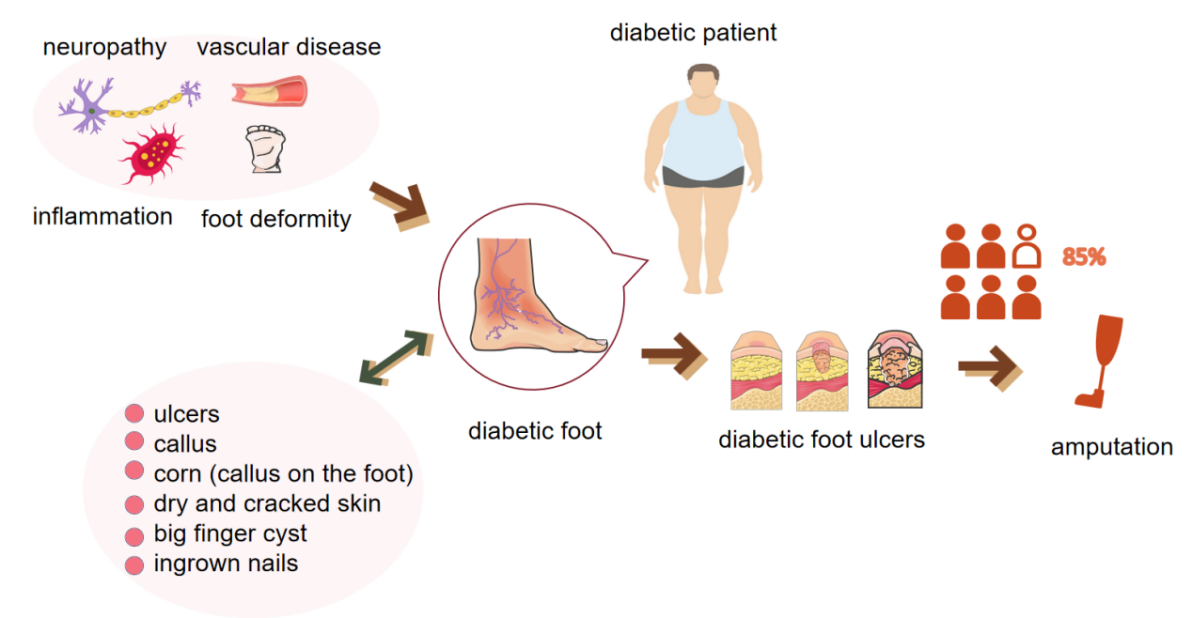


Fig1. Pathologic factors, clinical manifestations of the diabetic foot and its further progression to severe ulceration and amputation.


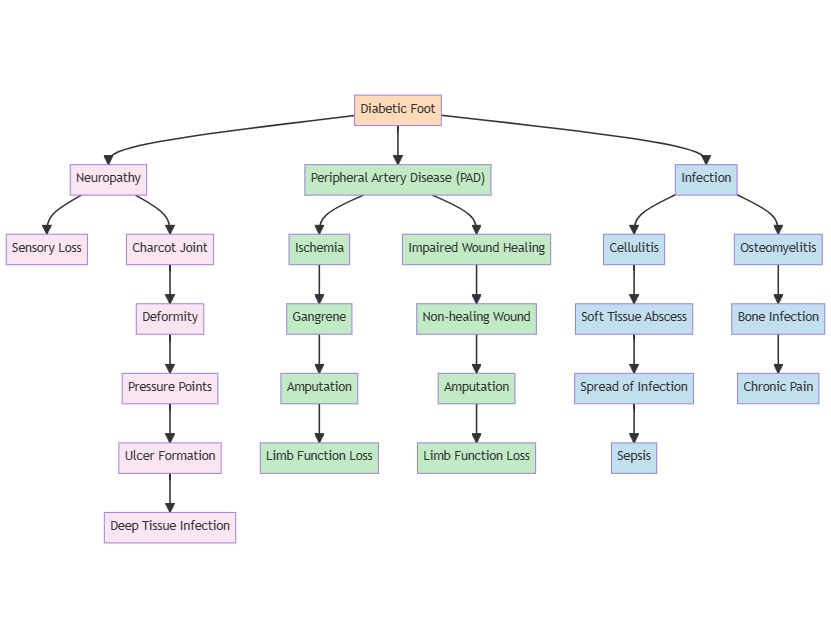


Figure 2: Pathogenesis and progression of complications in the diabetic foot

Neuropathy: Involves loss of sensation, Charcot joints, deformities, pressure points, ulcer formation and deep tissue infections.

Peripheral Artery Disease: involves ischemia, gangrene, amputation, loss of limb function, impaired wound healing, non-healing wounds, and re-amputation.

Infection: Involves cellulitis, soft tissue abscesses, spread of infection, sepsis, osteomyelitis, bone infections, and chronic pain.


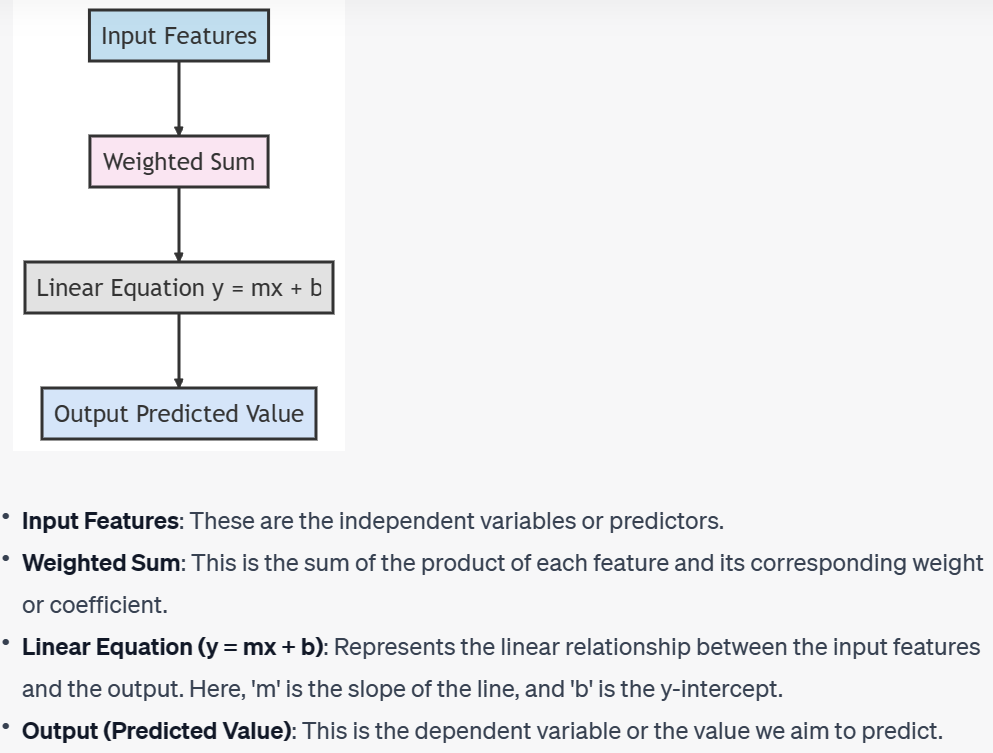


Figure 3: Mechanism diagram for linear regression.

Input Features: These are the independent variables or predictors.

Weighted Sum: This is the sum of the product of each feature and its corresponding weight or coefficient.

Linear Equation (y = mx + b): Represents the linear relationship between the input features and the output. Here, 'm' is the slope of the line, and 'b' is the y-intercept.

Output (Predicted Value): This is the dependent variable or the value we aim to predict.


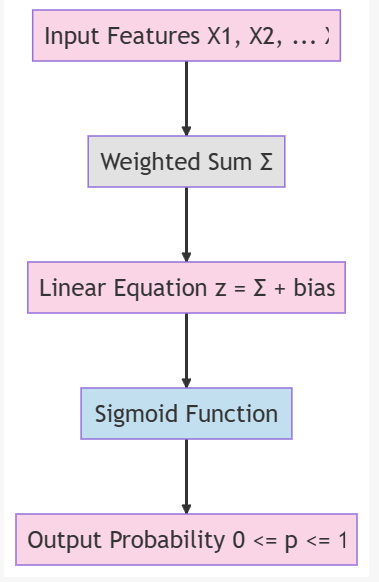


Figure 4: Mechanism diagram for logistic regression.

Input features (X1, X2, ... Xn): these are the independent variables or predictors used as inputs to the logistic regression model.

Weighted Sum (Σ): this step involves multiplying each input feature with its corresponding weight and adding them up. This is a linear combination of the inputs.

Linear equation (z = Σ + deviation): the weighted sum is then added to the deviation term to obtain the final linear equation.

Sigmoid Function: The output of a linear equation is passed through a sigmoid function, which maps any input to a value between 0 and 1. This function gives the model its probabilistic properties.

Output Probability (0 <= p <= 1): The output of a Sigmoid function is a probability value between 0 and 1. This probability indicates the likelihood that the given input data belongs to a particular category.


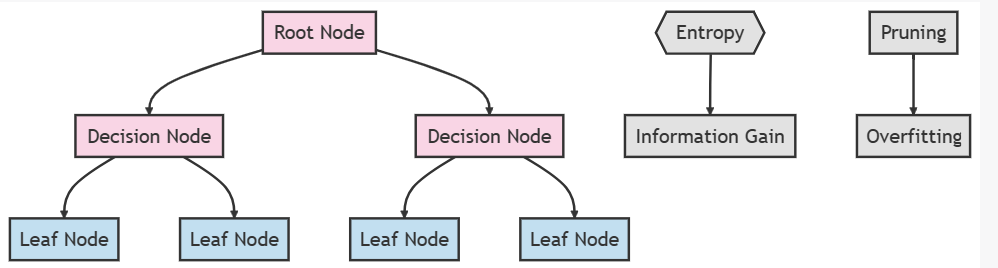


Figure 5: Mechanism diagram of the decision tree.

Root Node: This is the starting point of the decision tree, which uses the entire data set to make the first decision.

Decision Nodes: At these nodes, the data is further segmented based on specific attribute values.

Leaf nodes: These are the final outputs or decision results of the decision tree.

Entropy: This is a measure of randomness or uncertainty in a data set.

Information Gain: This is the criterion for selecting an attribute that tells us which attribute gives the most information, or which attribute is most effective in categorizing the data.

Pruning: This is a technique to reduce the size and complexity of the tree to avoid overfitting.

Overfitting: Overfitting occurs when the model is too sensitive to noise or random fluctuations in the training data. This means that the model may not perform well on new data.


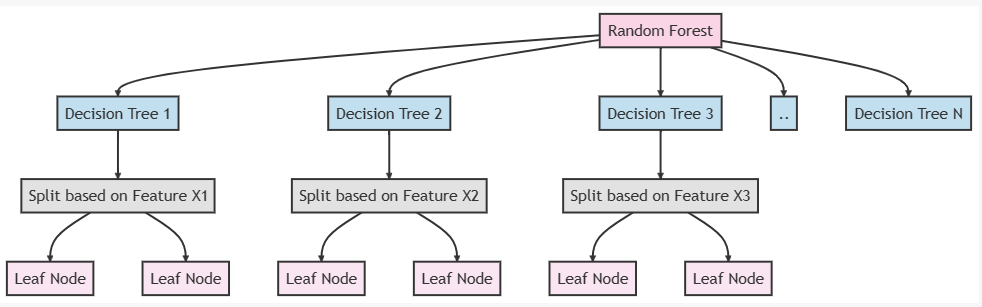


Figure 6: Mechanism diagram of random forest

Random Forest: this is an integrated learning method that combines multiple decision trees to produce more accurate and stable predictions. Each tree in the forest is constructed using a subset of the training data and features.


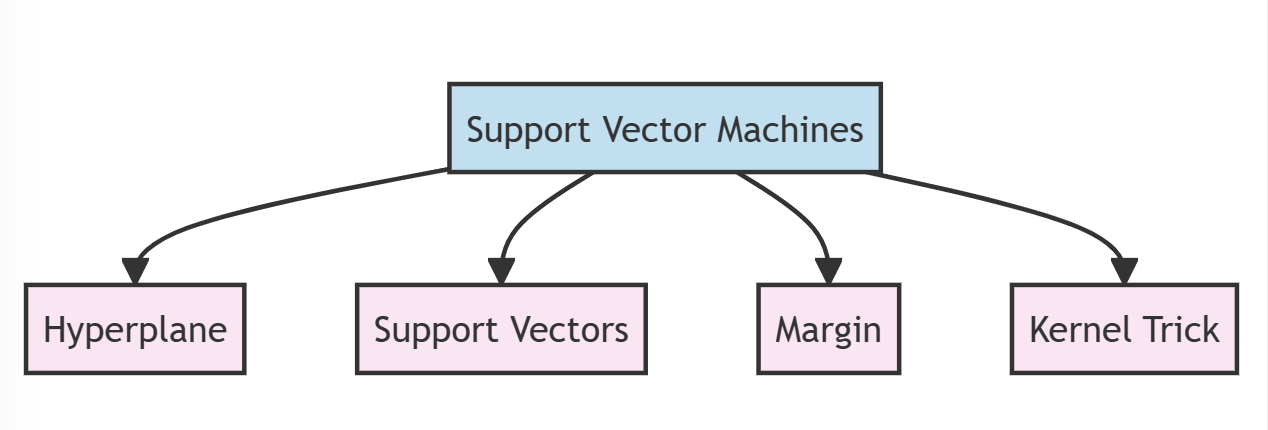


Figure 7: Mechanism diagram of support vector machine.

Hyperplane: the decision boundary used by SVM to categorize data.

Support Vectors: The closest data points to the hyperplane that affect its position and orientation. Margin: the distance between the hyperplane and the nearest data point in the two classes. It is shown in light pink.

Kernel Trick: A technique used by SVMs to transform data into a higher dimensional space so that it can be linearly separated.


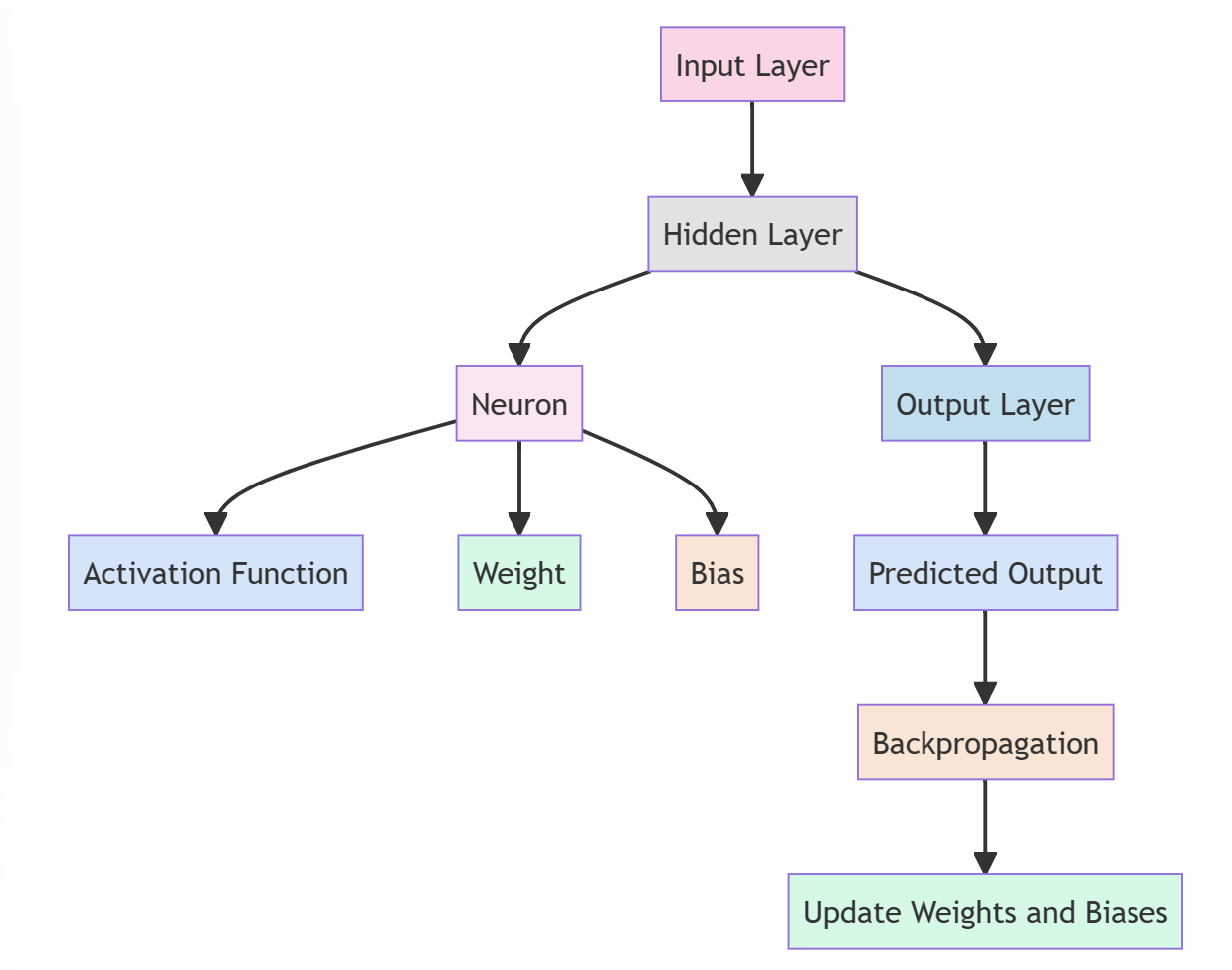


Figure 8: Mechanism diagram of neural network.

Input Layer: this is the initial layer of the data input network. It consists of neurons that receive input features and pass them to the next layer.

Hidden layers: these are the layers between the input and output layers. They process the input received from the input layer and transmit the processed information to the output layer. Depending on the complexity of the problem and the network architecture, the number of hidden layers and the number of neurons per layer may vary.

Output Layer: this is the final layer that produces results or predictions. It consists of neurons that provide the final output, which can be classification, regression values, etc.

Neurons: these are the basic computational units of a neural network. Each neuron receives inputs, processes them, and passes them through an activation function. Activation function: this function determines the output of the neuron.

Weights and biases: these are parameters of the neural network that are adjusted during training. The weights determine the strength of the connections between neurons, while the bias allows for shifts in the activation function.

Predicted Output: this is the result produced by the neural network after processing the input data.

Backpropagation: this is a training algorithm used to minimize the error between the predicted output and the actual output. It calculates the gradient of the error with respect to the network weights and adjusts the weights to minimize the direction of the error.

Updating weights and biases: based on the errors calculated during backpropagation, the weights and biases of the network are updated to improve the prediction in subsequent iterations.

Essentially, a neural network receives input data, processes it through multiple layers using weights and biases, and produces an output. During training, the network adjusts its weights and biases to minimize the error between its prediction and the actual output.


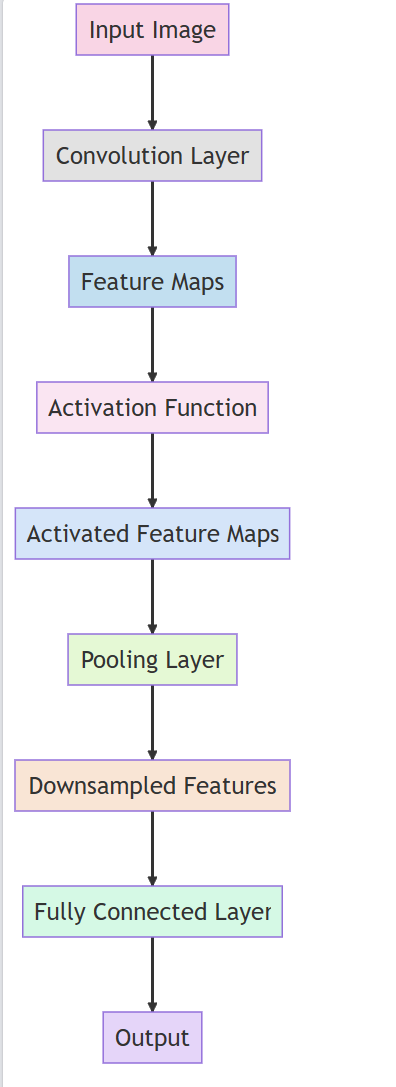


Fig. 9:Mechanism diagram of convolutional neural network.

Input Layer: The input layer receives the raw data.

Convolutional Layer: A convolutional layer is used to detect specific features in an image.

Activation Function): The activation function increases the nonlinearity and makes the model more robust.

Pooling Layer: Pooling layer is used to reduce the dimensionality of features while retaining their important information.

Fully Connected Layer: The Fully Connected Layer connects the learned features to the final output.

Output Layer: The output layer provides the final classification or regression results.

Feature Maps: Feature maps represent the features detected by the convolutional layer.

Downsampled Features: Downsampled features after pooling layer processing.


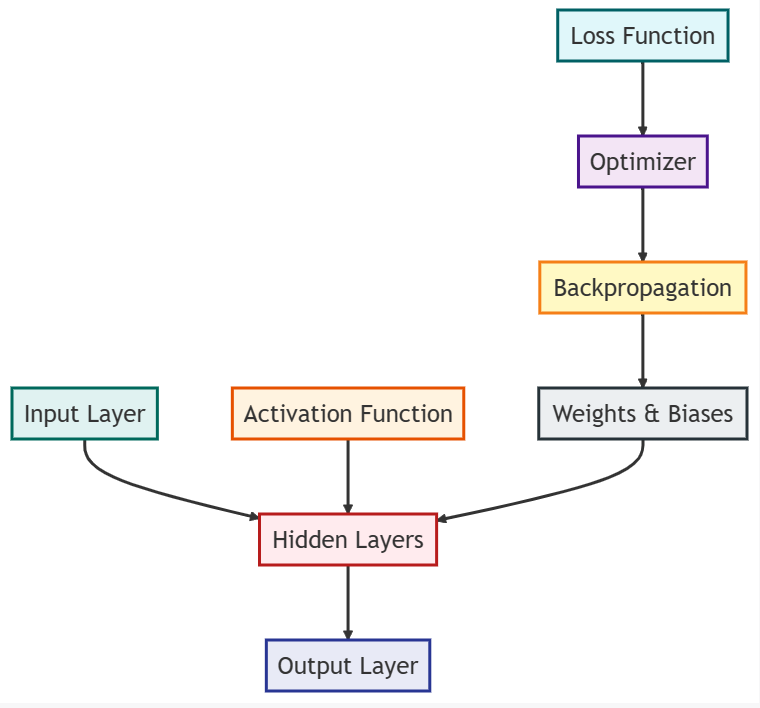


Fig. 10:Mechanism diagram of deep learning.

Input layer: the initial layer of data input to the neural network.

Hidden layers: layers between the input and output layers. They process the input and transfer the processed information to the output layer.

Output layer: the final layer that produces results or predictions.

Activation function: determines the output of a neuron. It adds nonlinearity to the network, allowing it to learn from errors and adapt, which is crucial for learning complex patterns.

Weights and biases: parameters of the neural network that are adjusted during training. The weights determine the strength of the connections between neurons, while the bias allows for shifts in the activation function.

Loss function: measures the difference between the predicted output and the actual output. The goal during training is to minimize this loss.

Optimizer: adjusts properties of the neural network, such as weights and learning rate, to minimize errors. Examples include SGD, Adam, and RMSprop.

Backpropagation: a training algorithm used to minimize errors by adjusting the weights and biases of the network.


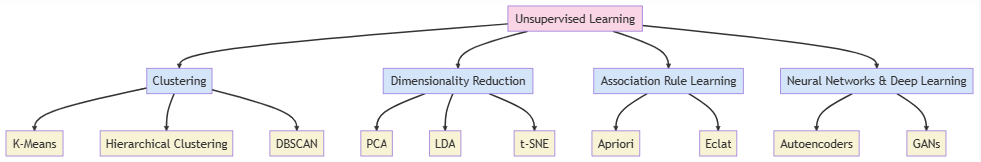


Figure 11: Classification of unsupervised learning and diagram of mechanisms.

Clustering: A method of grouping similar data points together based on certain characteristics.

K-means: an algorithm that divides data into K non-overlapping subsets.

Hierarchical Clustering: An algorithm that constructs a hierarchy of clusters by either bottom-up or top-down methods.

DBSCAN: A density-based clustering algorithm that combines points that are close to each other based on a distance measure.

Dimensionality reduction: A technique used to reduce the number of input variables in a data set.

Principal Component Analysis: a method used to emphasize variation and capture strong patterns in a data set.

Linear Discriminant Analysis: a method for finding linear combinations of features that separate two or more classes.

t-SNE: A nonlinear dimensionality reduction technique that is well suited for embedding high-dimensional data.

Association Rule Learning: A method for discovering interesting relationships between variables in large databases.

Apriori: An algorithm for identifying frequent itemsets in a dataset.

Eclat: a depth-first search algorithm for finding frequent itemsets.

Neural Networks and Deep Learning: a subset of machine learning using multi-layer neural networks.

Self-Encoder: A type of artificial neural network used to learn efficient encoding of unlabeled data.

Generative Adversarial Networks: a class of machine learning systems in which two neural networks compete with each other.


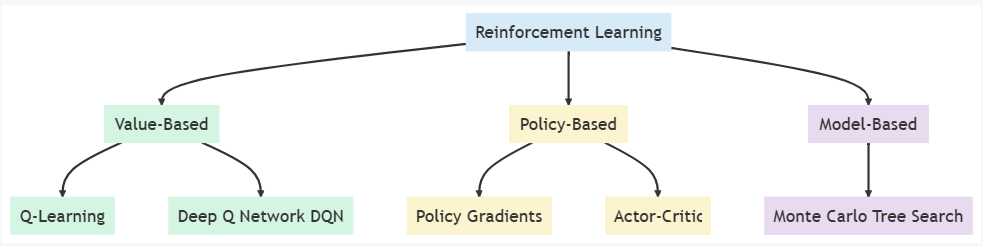


Figure 12: Classification and Mechanisms of Reinforcement Learning Map.

Value-based: A method of trying to find the best value function that indicates how good it is to be in a particular state.

Q-learning: an offline policy algorithm that learns the value of an action in a given state.

Deep Q Networks: combines Q learning with deep neural networks.

Strategy-based: A method that attempts to find the optimal strategy directly, without the need for a value function.

Strategy Gradient: an algorithm that optimizes the parameters of a strategy by following a gradient of higher rewards.

Actor-Critic: Combining Value-Based and Strategy-Based Approaches.

Model-based: An approach to modeling the environment.

Monte Carlo Tree Search: A search algorithm for decision making.

Table 1: Different application functions of different machine learning methods in diabetic foot research

| Category | Subcategory | Algorithms | Specific Features | Literature applications |
| --- | --- | --- | --- | --- |
| Supervised learning | regression (statistics) | linear regression | Simplicity and interpretability | (108) |
|  | categorization | Decision Trees and Random Forests | Interpretability;  Feature Selection:  Help researchers identify key biomarkers or risk factors. | (52, 53, 110, 112, 113) |
|  |  | support vector machine | Classification tasks:  Such as distinguishing diabetic foot from other foot disorders. | (52, 53, 104, 112) |
|  |  | logistic regression | Used to predict the risk of diabetic foot, e.g., based on the patient's age, gender, and history of diabetes. | (52, 112, 113) |
|  | Deep Learning and Neural Networks | Convolutional Neural Network | Used to analyze medical images of the foot, such as X-ray, MRI or ultrasound images, to detect early signs or complications of diabetic foot. | (25, 107, 109, 112) . |
| unsupervised learning | clustering | K-Means | subgroup analysis | (107) . |
| transfer learning |  |  | Enhancing model performance using pre-trained models from related fields, e.g., diabetic retinopathy, when diabetic foot data is limited | (103) . |
| Integrated Methods |  | Boosting | Stepwise optimization: an advantage when dealing with diabetic foot data with complex features and nonlinear relationships. | (25, 52, 107, 111, 112) |
